# NTRK1/TrkA Activation Overrides the G_2_/M-Checkpoint upon Irradiation

**DOI:** 10.3390/cancers13236023

**Published:** 2021-11-30

**Authors:** Christina Hassiepen, Aashish Soni, Ines Rudolf, Vivian Boron, Sebastian Oeck, George Iliakis, Alexander Schramm

**Affiliations:** 1 West German Cancer Center, Department of Medical Oncology, University Hospital Essen, University of Duisburg-Essen, 45147 Essen, Germany; christina.hassiepen@gmx.net (C.H.); inesrudolf@gmx.net (I.R.); vivian.boron@googlemail.com (V.B.); sebastian.oeck@uk-essen.de (S.O.); 2 Division of Experimental Radiation Biology, Department of Radiation Therapy, University of Duisburg-Essen Medical School, 45122 Essen, Germany; aashish.soni@uk-essen.de (A.S.); Georg.Iliakis@uk-essen.de (G.I.); 3 Institute of Medical Radiation Biology, University of Duisburg-Essen Medical School, 45122 Essen, Germany

**Keywords:** TrkA/NTRK1, irradiation, neuroblastoma, G_2_-checkpoint, cell cycle

## Abstract

**Simple Summary:**

Neuroblastoma (NB) is a solid childhood tumor and needs to be treated with multimodal therapy including radiation in advances stages. TrkA/NTRK1 expression is a hallmark of NB with excellent prognosis, but the impact of TrkA/NTRK1 on radiation response is largely unknown. Here, we report that human neuroblastoma cell lines engineered to express TrkA/NTRK1 in tightly controlled systems fail to activate the G2/M cell cycle checkpoint upon irradiation, which recapitulates the effects of ATM or ATR inhibition. Our findings point to a hitherto unrecognized TrkA/NTRK1-mediated wiring of the radiation response in NB cells.

**Abstract:**

High expression of the receptor tyrosine kinase TrkA/NTRK1 is associated with a favorable outcome in several solid tumors of childhood including neuroblastoma. During development, TrkA/NTRK1 governs migration and differentiation of neuronal precursor cells, while it is associated with mitotic dysfunction and altered DNA damage response, among others, in neuroblastoma. Here, we used human neuroblastoma cell lines with inducible TrkA/NTRK1 expression to mechanistically explore the role of TrkA/NTRK1 signaling in checkpoint activation after DNA damage induced by ionizing radiation (IR). TrkA/NTRK1 activated cells showed increased short-term cell viability upon IR compared to vector control cells. This was accompanied by a deficient G_2_/M-checkpoint at both low (1 Gy) and high doses (4 Gy) of IR. In a tightly controlled setting, we confirmed that this effect was strictly dependent on activation of TrkA/NTRK1 by its ligand, nerve growth factor (NGF). TrkA/NTRK1-expressing cells displayed impaired ATM and CHK1 phosphorylation, resulting in stabilization of CDC25B. In line with these findings, ATM or ATR inhibition recapitulated the effects of TrkA/NTRK1 activation on the IR-induced G_2_/M-checkpoint. In conclusion, we here provide first evidence for a previously unrecognized function of NTRK signaling in checkpoint regulation and the response to IR.

## 1. Introduction

Neuroblastoma NB comprises 8–10% of all childhood cancers, accounts for 15% of pediatric cancer deaths, and is the most common solid tumor in childhood [1]. The tumor derives from primitive sympathetic neural precursors and most often is found in the adrenal medulla [2]. A hallmark of NB is its highly heterogeneous disease outcome. Clinical courses range from spontaneous regression to the development of metastasis or relapse accompanied by therapy resistance and infaust prognosis [3]. Spontaneous tumor regression is frequently observed in infants below 18 months of age, even without chemotherapy [4]. On the other hand, the survival rate for children 18 months or older is only 40–50%, as neuroblastomas are often unresectable, metastatic, and require intensive multi-modal therapy [5]. Only about 1–2% of children with neuroblastoma have a genetic predisposition to the disease [6]. Hereditary neuroblastomas are caused by germline mutations in ALK (anaplastic lymphoma kinase, 75%) [7,8] or PHOX2B (paired-like homeobox 2B gene; 5%) [9]. Nevertheless, most neuroblastomas occur spontaneously with the underlying factors remaining largely unknown. The clinical heterogeneity of neuroblastoma is reflected by its variety of biological and genetic features. Important molecular features correlating with the differences in clinical outcome are telomerase activation [10], the status of the MYCN oncogene, which is amplified in about 20% of all NB [11], loss of heterozygosity at chromosomes 1p [12] or 11q [13], trisomy of 17q [14], and high expression of the neurotrophin receptor TrkB/NTRK2 [15], all of which are linked to unfavorable outcome [16]. Near-triploidy [17] and a high expression of TrkA/NTRK1 [18], on the other hand, are associated with more benign tumors that often regress spontaneously [19]. We and others have shown in in vitro models that activation of TrkA/NTRK1 by NGF induces reprogramming of signaling pathways by altering gene expression profiles, proliferation, and neuronal growth [20,21]. Expression of TrkA/NTRK1 has also been linked to heterotypic cell interactions enabling neuroblastoma cells to communicate with immune cells and stromal cells [22]. Moreover, gene expression profiling data also suggested that NB cells engineered to constitutively express TrkA/NTRK1 regulate factors associated with non-homologous end-joining. Gene expression analyses of DNA repair-factors revealed an up-regulation of the NHEJ-factor XRCC4 in SY5Y-NB cells upon ectopic expression of TrkA/NTRK1 [23], but the consequences of TrkA/NTRK1 on cell cycle regulation upon IR remained elusive.

It is known that several cancers acquire multiple defects in checkpoint signaling, among which the aberrant transition from G_2_ to M phase plays a crucial role [24]. Therefore, G_2_ checkpoint signaling modulation has been widely investigated in pre-clinical and clinical settings. Two of the master regulators of the G2 checkpoint are ATM and ATR, which function through activation of their respective downstream kinases, Chk1 and Chk2 and several other DNA-damage response proteins [25,26,27]. These key regulators of the G_2_ checkpoint have been identified as targets for inhibitor development and several of these inhibitors are now evaluated in clinical trials for several cancer entities. Ionizing radiation (IR) is a promising treatment modality in many tumor types with altered DNA double strand breaks (DSBs) repair. DSB repair pathways are shown to cooperate with DNA damage cell cycle checkpoints [28,29] to safeguard genomic stability when cells are exposed to IR. Thus, inhibition of checkpoint proteins in combination with IR is deemed an attractive combinatorial treatment regimen. Understanding molecular circuits governing the checkpoint response is crucial for defining patient populations, which will benefit from such treatment modalities [30].

As current therapeutic regimens of NB also include radiotherapy, we here evaluated the response of neuroblastoma cells to IR as a function of TrkA/NTRK1 expression, which is a master regulator of NB biology. For this purpose, we used existing [21] and newly established pre-clinical models engineered to conditionally express TrkA/NTRK1 to evaluate differential responses when cells were irradiated in the presence or absence of TrkA/NTRK1 activation. Our findings imply that TrkA activation is correlated with a defective G_2_/M checkpoint upon irradiation. We demonstrate that TrkA/NTRK1 activation overrides the G_2_/M checkpoint by down-regulating both, ATM-Chk2 and ATR-Chk1 signaling.

## 2. Results

### 2.1. Inducible Expression of TrkA/NTRK1 in Human Neuroblastoma Cell Lines Increases Short-Term Survival upon Ionizing Radiation (IR)

**Figure 1:**
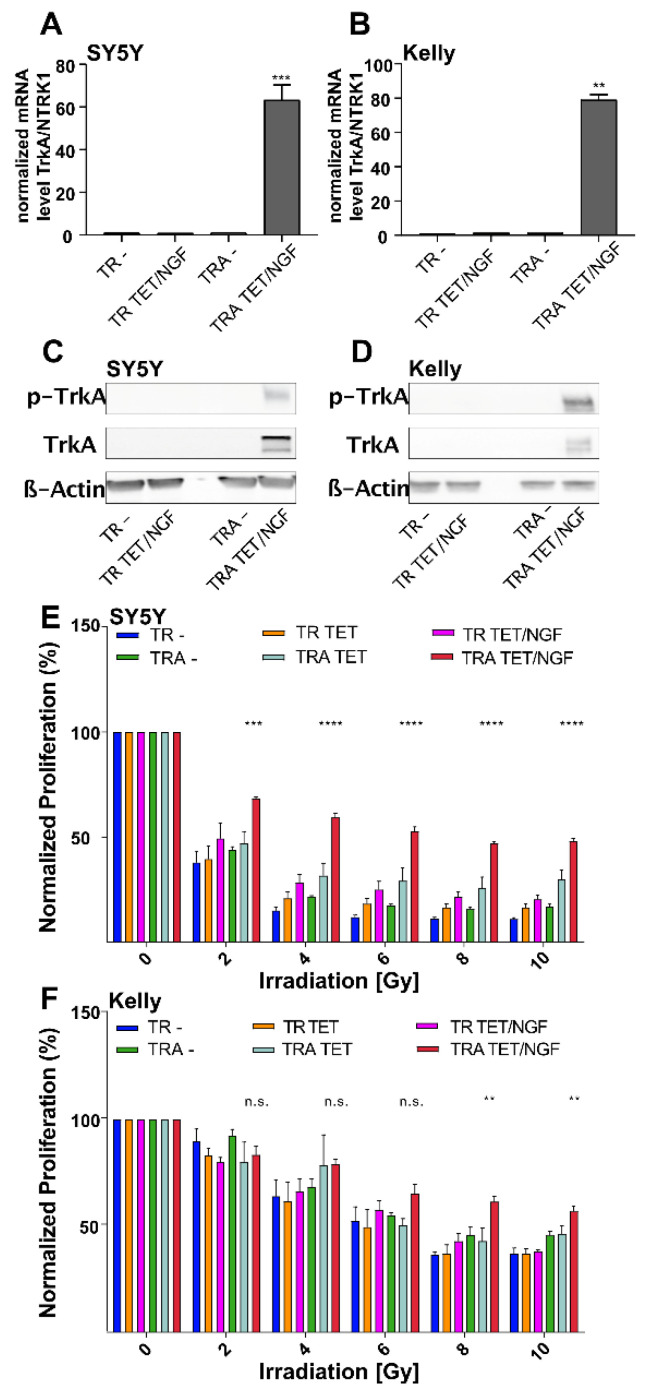
Inducible expression and activation of TrkA/NTRK1 in SY5Y-TRA and Kelly-TRA cells and the impact on proliferation upon ionizing radiation. (**A**,**B**) SY5Y and Kelly cells were engineered to express TrkA/NTRK1 upon addition of tetracycline (TET). The suffix “-TR” indicates the presence of the TET-repressor only, while the suffix “-TRA” refers to cells harboring the TR and the TET-responsive element controlling TrkA/NTRK1 expression. Shown are mRNA levels of TrkA/NTRK1 in the cell lines indicated. Data were obtained by qPCR and normalized to the expression of the house keeping gene, GAPDH (*n* = 3 from three independent experiments). (**C**,**D**) Western blot showing protein expression of TrkA/NTRK1 (labelled “TrkA”) as well as TrkA/NTRK1 activation (indicated by receptor phosphorylation, labelled “p-TrkA”). TrkA/NTRK1 activation is only observed in SY5Y-TRA and Kelly-TRA cells upon addition of the TrkA/NTRK1-ligand, NGF. Original Western Blot data are shown in Supplementary Figure S1 (**E**,**F**) TrkA/NTRK1 activation results in significantly higher cell proliferation upon IR compared to parental cells and controls. Asterisks indicate significant differences between irradiated cells with and without TrkA/NTRK1 activation as indicated (**** = *p* < 0.0001, *** = *p* < 0.001, ** = *p* < 0.01). Irradiation was performed at different dose intensities (2, 4, 6, 8 or 10 Gy). Proliferation was evaluated by MTT assay after 96 h. Error bars show the SEM from three independent experiments.

To study the immediate and long-term effects of TrkA/NTRK1-signaling upon exposure to IR, TrkA/NTRK1-negative human neuroblastoma cell lines, SY5Y and Kelly, were engineered to conditionally express TrkA/NTRK1 upon addition of tetracycline (TET) and designated SY5Y-TRA and Kelly-TRA, respectively. We first validated that NTRK1 mRNA expression is restricted to SY5Y-TRA and Kelly-TRA cells exposed to TET (Figure 1A,B). Furthermore, activation of TrkA/NTRK1 in SY5Y- TRA and Kelly TRA was only observed upon TrkA/NTRK1 activation by its ligand, NGF (Figure 1C,D).

To evaluate the consequences of the response to IR depending on TrkA/NTRK1 expression, SY5Y-TRA and Kelly-TRA cells as well as controls were exposed to different doses of IR. Activation of TrkA/NTRK1 resulted in residual proliferation activity that was significantly higher as compared to controls (Figure 1E,F).

### 2.2. TrkA/NTRK1-Activation Impacts on Cell Cycle Distribution and Induces Impaired G_2_-Arrest in Irradiated Neuroblastoma Cells

**Figure 2:**
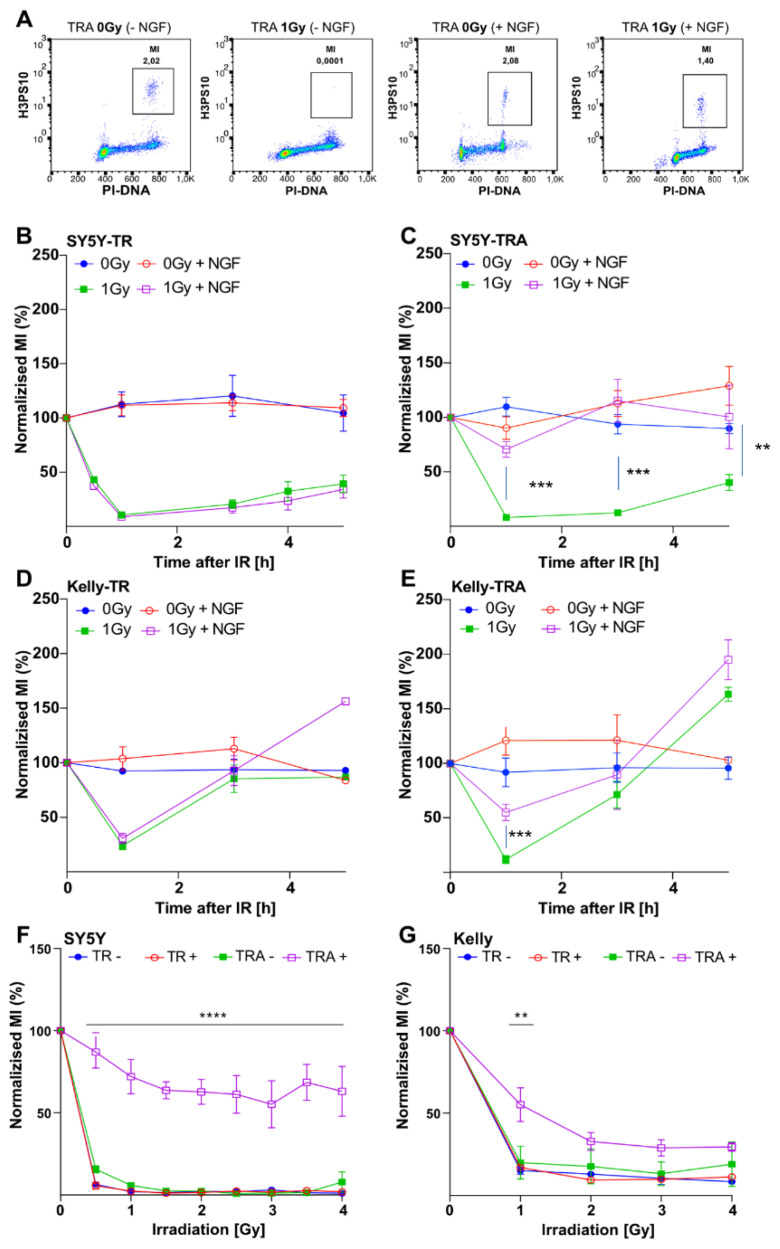
TrkA/NTRK1 activation abrogates the G_2_-checkpoint upon IR and induces a dose-independent G_2_ checkpoint defect in both SY5Y-TRA and Kelly-TRA cells. (**A**) Representative dot plots indicate the mitotic cell fraction (mitotic index [MI], marked by squares), which was defined as the number of H3pS10-positive cell divided by the total number of cells at 1 h post IR. (**B**) SY5Y-TR cells and (**D**) Kelly-TR cells were exposed to IR (1 Gy) and the MI determined at time points indicated. (**C**) SY5Y-TRA cells and (**E**) Kelly-TRA cells along with their respective controls were exposed to IR (1 Gy) and their MI was determined at time points indicated. (**F**,**G**) Analyses of the mitotic index of SY5Y and Kelly cells after irradiation with IR doses as indicated. Asterisks indicate significant differences between irradiated cells with and without TrkA/NTRK1 activation as indicated (**** = *p* < 0.0001, *** = *p* < 0.001, ** = *p* < 0.01). Error bars show the SEM from three independent experiments.

To explore underlying reasons for the TrkA/NTRK1-mediated effects on short-term proliferation, we analyzed changes in cell cycle distribution in SY5Y-TRA and Kelly-TRA cells by flow cytometry after exposing cells to IR. Analyses of cells irradiated during the S-phase of the cell cycle revealed that activation of TrkA/NTRK1 resulted in an increased fraction (>10%) of cells in G_2_-phase at all time points analyzed upon high-dose IR (4 Gy) (Supplementary Figure S2), but not at 1 Gy (Supplementary Figure S2). In the absence of TrkA/NTRK1-expression, no cells were observed in G_2_-phase at 4 h post-IR in the high-dose setting (4 Gy), while low-dose irradiation did not affect the fraction of cells in G_2_ irrespective of TrkA/NTRK1 expression and activation. To further discriminate mitotic cells from cells in G_2_ phase, SY5Y-TRA and Kelly-TRA cells were again irradiated and stained for the mitotic marker, H3pS10. The fraction of H3pS10-positive cells was then counted and used to calculate the mitotic index (MI, Figure 2A). In cells without TrkA/NTRK1 activation, the MI decreased to almost undetectable levels in SY5Y cells (SY5Y-TR) and was found reduced by >50% in Kelly-TR cells, in line with an intact G_2_-checkpoint upon low-dose (1 Gy) IR (Figure 2B,D). By contrast, the MI of cells with TrkA/NTRK1 expression and activation was significantly elevated (Figure 2C,E), indicating an impaired G_2_ checkpoint in these cells. Notably, this effect was strictly dependent on TrkA/NTRK1 activation by NGF. To better understand, if the cell cycle phase had an impact on the checkpoint response, we additionally labelled cells with EdU, which stains cells in S phase. Cells irradiated in S phase presented with a strong G2 arrest (Supplementary Figure S3). This also explains the increased proportion of G2/M phase cells at 4 h after irradiation. To further characterize the effect of dose intensity on checkpoint response, MIs were determined in a dose range between 0.5 to 4 Gy for SY5Y-TRA and Kelly-TRA cells (Figure 2F,G). At all doses analyzed, activation of TrkA/NTRK1 induced a G_2_ checkpoint defect in both SY5Y-TRA and Kelly-TRA cells indicating that the TrkA/NTRK-mediated checkpoint defect is independent of the applied IR dose. Taken together, different layers of evidence suggested a less stringent, dose-independent G_2_-checkpoint upon irradiation induced by TrkA/NTRK1 activation in neuroblastoma cells in vitro.

To check whether an IR-induced G_2_ checkpoint defect is specific for TrkA/NTRK1 or is a general consequence of tyrosine kinase activation in cancer cells, we used EGFR-overexpressing lung cancer cells, A549 and H1975, as a control. Activation of EGFR in A549 and H1975 cells was achieved by addition of the EGFR-ligand, EGF, 24 h before exposure to 1 Gy. In both cell lines, IR strongly decreased the MI, indicating an intact G_2_ checkpoint irrespective of EGFR activation (Supplementary Figure S4). These results excluded an impaired G_2_ checkpoint as a generic response of tyrosine kinases activation upon irradiation.

**Figure 3:**
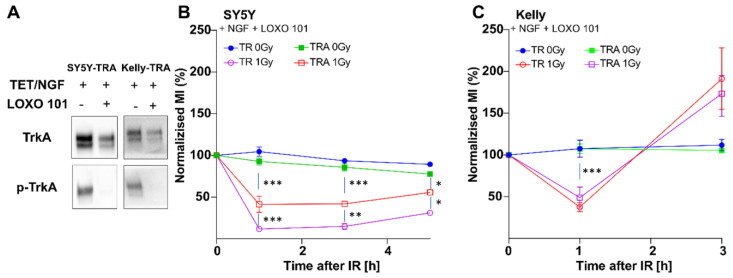
TrkA/NTRK1 inhibition abrogates the TrkA-mediated effect on the G2 checkpoint upon irradiation in SY5Y and Kelly cells (**A**) Expression and activation of TrkA/NTRK1 in SY5Y-TRA and Kelly-TRA cells with and without LOXO 101 treatment. Original Western Blots have been included as Supplementary Figure S5. Activation of TrkA/NTRK1 in SY5Y cells (**B**) or Kelly cells (**C**) was achieved by addition of NGF and cells were treated with the Trk-Inhibitor LOXO 101 prior to IR. MI values were normalized to the respective unirradiated controls. Asterisks indicate significant differences between experimental groups as indicated (*** = *p* < 0.001, ** = *p* < 0.01, * = *p* < 0.05). Error bars show the SEM from 3–5 independent experiments.

To demonstrate that the observed checkpoint defect was dependent on TrkA/NTRK1 activation, cells were again irradiated in the presence of absence of the specific Trk-inhibitor, LOXO 101, which completely abolished phosphorylation of NTRK1 in both, SY5Y-TRA and Kelly-TRA cells (Figure 3A). Addition of LOXO-101 prior to IR restored the G_2_ checkpoint response in SY5Y and Kelly cells with TrkA/NTRK1 activation indicated by a significant decrease of the number of cells in mitosis (Figure 3B,C). Notably, LOXO101 treatment could not fully restore the checkpoint response in SY5Y-TRA cells upon TrkA/NTRK1 activation, while there was no significant difference between Kelly-TR and Kelly-TRA cells with respect to G2 checkpoint activation. Nevertheless, these findings suggest that TrkA/NTRK1 activation is causally involved in the impaired G_2_/M-checkpoint upon IR.

### 2.3. Inhibition of Cell Cycle Checkpoint Proteins Recapitulate the Effect of TrkA Activation on the G2 Checkpoint Response

**Figure 4:**
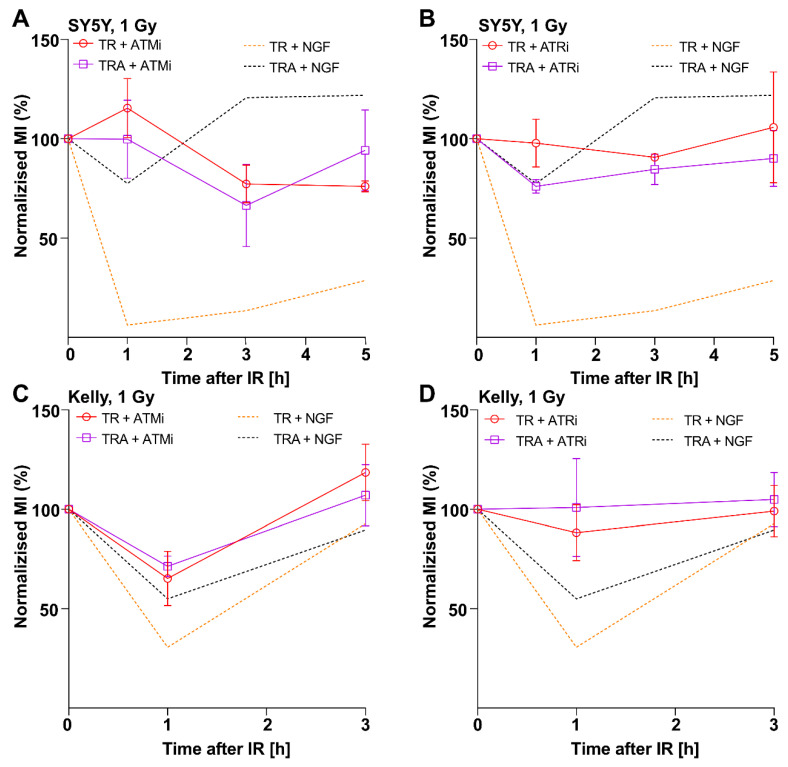
Inhibition of ATM- or ATR-signaling abrogates the G2 checkpoint upon IR. (**A**,**C**) SY5Y-TRA and Kelly-TRA cells with and without activation of TrkA/NTRK1 by NGF were exposed to an ATM inhibitor (ATMi, KU-55933) and irradiated with 1Gy. After irradiation, the mitotic indices (MI) were determined. (**B**,**D**) SY5Y-TRA and Kelly-TRA cells with and without activation of TrkA/NTRK1 by NGF were exposed to an ATR inhibitor (ATRi, VE821) and irradiated with 1Gy. After irradiation, the mitotic indices (MI) were determined. In all these experiments, cells containing the TET repressor only (SY5Y-TR, Kelly-TR) served as controls.

ATM and ATR are central pillars of the G_2_ checkpoint response, and their function is essential for activation of cell cycle checkpoints. We therefore asked whether G_2_ checkpoint defects in TrkA/NTRK1 activated cells depend on suppression of ATM and ATR signaling. To address this issue, we first analyzed the impact of specific ATM and ATR inhibitors on G_2_ checkpoint response in cells with or without TrkA/NTRK1 activation. Inhibition of either ATM by KU-55933 or ATR by VE821 abrogated the G_2_ checkpoint in irradiated SY5Y-TRA and Kelly-TRA cells irrespective of activation of TrkA/NTRK1 (Figure 4A–D). This suggests that response to IR-mediated G_2_-arrest in SY5Y and Kelly cells depends on the function of signaling downstream of both, ATM and ATR.

**Figure 5:**
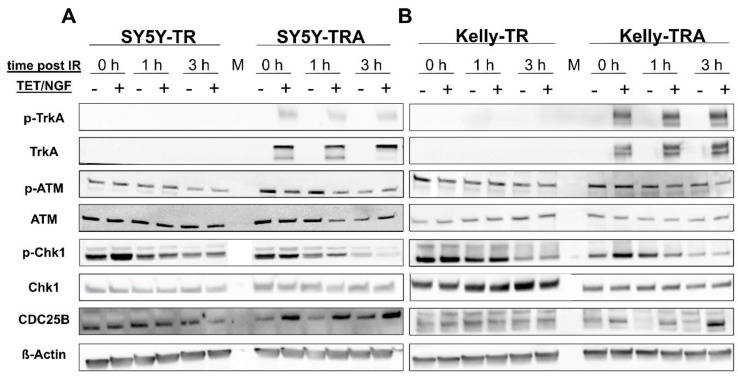
The ATM/ATR DNA-damage signaling pathway is inhibited by TrkA/NTRK1 activation in SY5Y and Kelly cells. Representative Western blots showing expression and phosphorylation of TrkA/NTRK1, ATM, Chk1, and CDC25B. Expression of ß-Actin served as loading control. Original Western Blot data are provided as Supplementary Figure S8 (**A**) SY5Y and (**B**) Kelly TR and TRA cells. Cell were exposed to irradiation with 1 Gy and analyzed at time points thereafter as indicated.

To analyze the impact of TrkA/NTRK1 expression on checkpoint signaling proteins, we determined expression and activation levels of key proteins involved in the G_2_/M-checkpoint response. Protein samples were collected at 0, 1, and 3 h post IR from both SY5Y-TRA and Kelly-TRA cells and controls with and without TrkA/NTRK1 activation after exposure to IR (1Gy). We observed reduced p-ATM and p-Chk1 levels in cells with activation of TrkA/NTRK1 (Figure 5A,B); however, this did not reach statistical significance (Supplementary Figure S6). Interestingly, Chk1 and ATM expression levels remain unchanged in cells with TrkA/NTRK1 activation upon IR. Additionally, stabilization of CDC25B, which is a requirement for entry into mitosis, could be confirmed in both cell lines, SY5Y and Kelly, upon TrkA/NTRK1 activation. In addition, we also validated on-target effects of ATMi, ATRi, and LOXO-101 on checkpoint signaling proteins including Chk1 upon irradiation (Supplementary Figure S7), confirming that the canonical signaling of ATM and ATR is active in our model systems. Taken together, these results support the notion that the ATM-ATR-Chk1 pathway is a downstream target of TrkA/NTRK1 that governs the G_2_ checkpoint response upon irradiation.

## 3. Discussion

Multimodal therapy of high-risk neuroblastoma includes irradiation as a standard treatment option. It is presently unclear, which modifiers of the radiation response would predict responses or resistance of neuroblastoma to irradiation. Of note, high-risk neuroblastomas are often associated with copy number alterations of individual chromosomes, most prominently loss of 1p and 11q as well as 17q gain [12,14,31]. By contrast, more benign disease courses, frequently associated with TrkA/NTRK1 expression, are characterized by near-triploid tumor genomes suggesting defects in mitotic control rather than genomic instability. We therefore established inducible TrkA/NTRK1 expression in two human neuroblastoma cell lines, SY5Y and Kelly, to dissect the impact of TrkA/NTRK1 on cell cycle regulation and checkpoint responses. In this tightly controlled system, we could show that activation of TrkA/NTRK1 significantly enhanced short term proliferation after irradiation compared to parental cells and controls. In-depth analyses of cell cycle distribution indicated that the G2/M checkpoint response of SY5Y and Kelly cells was dysfunctional after irradiation with either 1 Gy or 4 Gy in cells irradiated during G2 phase of the cell cycle. By contrast, TrkA/NTRK1-negative cell lines and cells without TrkA/NTRK1 activation had an intact G_2_-checkpoint indicated by the absence of H3pS10-positive cells at 1 h post-IR. TrkA/NTRK1-activation led to a significantly higher proportion of mitotic cells after irradiation so that the fraction of cells in mitosis was still at 50% compared to unirradiated controls at 1 h post IR. On the other hand, cells irradiated in S-phase displayed a significant G2 checkpoint arrest as indicated by accumulation of EdU-positive cells in G2 phase (Supplementary Figure S3). It has been shown that ATM-deficient cells have two distinct types of G2 checkpoint responses depending on the cell cycle phase they were in during IR [32]. This resulted in significant G2 checkpoint arrest for cells irradiated in S phase. Similarly, we could recently demonstrate that the checkpoint responses in cells deficient for homologous recombination also depend on the cell cycle phase at the time point of IR [29]. These findings are in line with the observed G2/M checkpoint defect upon TrkA/NTRK1 activation that is specific for irradiated G2 cells. The underlying processes regarding DNA repair mechanisms still need to be elucidated. However, two lines of evidence support the hypotheses that the observed G_2_/M checkpoint defect is a specific consequence of TrkA/NTRK1 activation. First, inhibition of TrkA/NTRK1 abrogated the mitotic entry of neuroblastoma cells with activated TrkA/NTRK1. Second, no effect of receptor tyrosine kinase activation on the checkpoint response was observed in EGFR-dependent lung cancer cell lines upon addition of EGF. Trk-specificity of this effect on checkpoint regulation is further corroborated by the finding that Trk-inhibition by a small molecule inhibitor, LOXO 101, abrogates the TrkA/NTRK1-mediated survival benefit upon irradiation in SY5Y and Kelly cells. However, these cells differ in the status of the MYCN oncogene: SY5Y is MYCN-normal, while Kelly cells are MYCN-amplified. The latter is known to be associated with Chk1 activation and MYCN-amplified cells are exquisitely sensitive to Chk1 inhibition [33]. Our results further suggest that the ATM/ATR signaling is intact in SY5Y and Kelly cells, as both ATM or ATR inhibition recapitulated the phenotype of TrkA/NTRK1 activation. It has been previously reported that PI3K signaling is a canonical pathway regulated by TrkA/NTRK1. Constitutively active PI3K enabled cells to override the DNA damage-induced G_2_/M-checkpoint [34]. The mechanism by which over-activation of PI3K inhibits G_2_-arrest may involve PKB/AKT-mediated inactivation of Chk1 through phosphorylation at Serine 280, thus impairing phosphorylation of Cdc25 and preventing inhibition of the Cyclin B1/Cdc2 complex [35]. Of note, TrkA/NTRK1 activation facilitated stabilization of CDC25B upon IR, which is a prerequisite for entry into mitosis [36], and this was not observed in control cells without TrkA/NKTR1 activation. In summary, to the best of our knowledge this is the first report causally linking TrkA/NTRK1 activation and regulation of the G_2_-checkpoint in neuroblastoma cells. It is tempting to speculate that the impaired G2/M checkpoint could at least in part explain the genomic instability frequently observed in primary TrkA/NTRK1 expressing neuroblastoma.

## 4. Materials and Methods

### 4.1. Cell Lines and Culture Conditions

Human neuroblastoma cell lines, SY5Y and Kelly TR were first transfected with pcDNA6/TR, containing a tetracycline repressor gene and the resulting cell lines were designated SY5Y-TR and Kelly-TR. Next, pT-REx-DEST30 (Invitrogen, Carlsbad, CA, USA) containing NTRK1 cDNA was transfected into SY5Y-TR and Kelly-TR to give rise to SY5Y-TRA and Kelly-TRA, respectively. Single cell clones were selected upon addition of blasticidin and G418 to the medium. NTRK1/TrkA expression was induced by tetracycline for 24 h before TrkA was activated by addition of NGF (100 ng/mL, R&D Systems, Minneapolis, NE, USA) as described [21]. A549 and H1975 cells are derived from non-small cell lung cancers characterized by high expression of EGFR [37]. In H1975, EGFR is constitutively active due to an activating mutation in the tyrosine kinase domain (L858R), while a second mutation (T790M) confers resistance to EGFR inhibitors [38]. All cell lines were grown in RPMI supplemented with 10% FBS and 5% PenStrep. Cells were incubated at 37 °C in a humidified atmosphere with 5% CO_2_ and 95% air.

### 4.2. Inhibitors and Antibodies

The specific ATM inhibitor (ATMi) 2-morpholin-4-yl-6-thianthren-1-yl-pyran-4-one (Calbiochem, KU-55933) was dissolved at 10 mM in dimethyl sulfoxide (DMSO) (Sigma-Aldrich), and used at 10 μM. The specific ATR inhibitor (ATRi) 3-amino-6-[4-(methylsulfonyl)phenyl]-*N*-phenyl-2-pyrazinecarboxamide (VE821, Haoyuan Chemexpress) was dissolved in DMSO at 10 mM, and used at 5 μM. Inhibitors were added 1 h before irradiation and maintained during the whole experimental procedure. Larotrectinib sulfate (LOXO 101) is a Trk-Inhibitor (Selleckchem) was dissolved at 1 mM in dimethyl sulfoxide (DMSO) (Sigma-Aldrich) and was used at 1.89 μM final concentration. All primary antibodies were obtained from Cell Signalling (CS) and used in a dilution of 1:1000 for Western Blot experiments (ATM, CS, #2873; p-ATM Ser1981, CS, #5883; TrkA, CS, #2510; p-TrkA/B Tyr785/Tyr816, CS, #4621; Chk1, CS, #2360; p-Chk1 Ser317, CS, #12302; CDC25B, CS, #9525). Anti-H3pS10 antibody was obtained from Abcam (ab5176).

### 4.3. Irradiation

Irradiations were carried out with an X-ray machine (GE-Healthcare, Chicago, IL, USA) operated at 320 kV, 10 mA with a 1.65 mm Al filter (effective photon energy approximately 90 kV), at a distance of 50 cm, and a dose rate of approximately 1.3 Gy/min. Dosimetry was performed with a survey meter (PTW) and/or a chemical dosimeter, which were used to calibrate an infield ionization monitor [29].

### 4.4. Viability Assay

For monitoring cell viability, 2000 cells were plated in a 96-well plate. Induction and activation of TrkA/NTRK1 by TET or TET/NGF prior to irradiation (0.5–4 Gy) was achieved as described [21]. After incubation for an additional 48 h, cell viability was analyzed using the 3-(4,5-dimethylthiazol-2-yl)-2,5-diphenyltetrazolium bromide (MTT) assay by quantitating the absorption at 565 nm in a plate reader (Bio-Rad Hercules, CA, USA) as described [20].

### 4.5. Calculation of the Mitotic Index Using Histone H3-pS10 (H3pS10) and PI Staining

Cells were harvested at 0, 1, 3, and 5 h after IR and fixed in 70% ethanol at—20 °C. After fixation, cells were resuspended in 1 mL of 0.25% Triton X100 in phosphate-buffered saline (PBST) prior to incubation in H3pS10 antibody (Abcam, 1:5000, Lot GR3250710-1) and subsequently washed according to the manufacturer’s recommendations. Detection was achieved using a secondary antibody coupled to Alexa Fluor 488 (Invitrogen, 1:300, Lot 2156521, in PBS-T) and addition of propidium iodide (PI, 4 µg/mL). Samples were analysed in a flow cytometer (Gallios, Beckman Coulter, Brea, CA, USA) and the fraction of mitotic cells determined by Kaluza (Beckman Coulter) and FlowJo software (BD Biosciences). To calculate the mitotic index, the number of H3pS10 and PI positive cells was normalized to the total number of cells.

### 4.6. Western Blot Analyses

Samples were prepared by solubilizing cells in RIPA buffer prior to loading on 4–15% SDS gels. Gels were run in a gel chamber for 15 min at 100 V and then for 45 min at 120 V. The proteins were transferred from to a nitrocellulose membrane using the Trans-Blot Turbo Transfer System (Bio-Rad). Prior to blocking, membranes were washed three times in TBS-T and blocking took place for 1 h with 5% BSA in TBS-T. The membrane was washed again with TBS-T and incubated overnight at 4 °C in the presence of the primary antibody. The antibody solutions consisted of 1% BSA in 5 mL TBS-T and a dilution of 1:1000 of the respective antibody listed above. Detection was achieved using “ECL Prime Western-Blotting Detection” reagents (GE Healthcare) and the ChemiSmart Imaging System (Vilber Lourmat, Collégien, France) and protein expression was quantified using the Fiji software (https://fiji.sc/ (Accessed on 25 October 2021)).

### 4.7. EdU Incorporation and Detection

Cells were pulse-labeled with 10 μM of 5-Ethinyl-2´-deoxyuridine (EdU) for 15 min and then irradiated and processed for flow cytometry. Briefly, cells were collected by trypsinization and permeabilized by incubating the cell pellets for 2 min in ice-cold PBS containing 0.2% Triton™ X-100 on ice. Cells were spun down and pellets were fixed for 15 min with 3% PFA plus 2% sucrose dissolved in PBS. Cells were blocked with PBG blocking buffer overnight at 4 °C. EdU signal was developed using an EdU staining kit (Thermo Fisher Scientific, Waltham, MA, USA) according to the manufacturer’s instructions. Finally, DNA was stained using Propidium iodide plus RNAase solution for 15 min at RT. Two-parameter FACS analysis was carried out with a Gallios flow cytometer (Beckman Coulter) and quantitated using Kaluza software 1.3 (Beckman Coulter, Brea, CA, USA).

### 4.8. Statistical Analyses

Results are expressed as mean ± SEM. Statistical significance between experimental groups were determined by *t*-test or, when means of three or more groups were compared, by two-way ANOVA followed by Bonferroni’s correction for multiple testing. Data analysis was performed with Prism 8.4.3 software (GraphPad, San Diego, CA, USA). The significance of differences between individual measurements is indicated by * symbol: * *p* < 0.05, ** *p* < 0.01, *** *p* < 0.001, n.s. non-significant.

## 5. Conclusions

In conclusion, we here provide first evidence for a previously unrecognized function of NTRK signaling in checkpoint regulation and the response to IR.

## Data Availability

Raw data are available in the Supplemental Material accompanying this manuscript.

## Supplementary Materials

The following are available online at https://www.mdpi.com/article/10.3390/cancers13236023/s1, Figure S1. Western Blots corresponding to Figure 1C,D including molecular weight markers. Figure S2. Activation of TrkA/NTRK1 increases the fraction of cells in G_2_/M phase after high-dose irradiation (4 Gy) in SY5Y-TRA. The cell cycle was analyzed by FACS and PI-staining in SY5Y cells with and without TET/NGF treatment. Cells were irradiated with (A) 1 Gy or (B) 4 Gy. Error bars show the SEM from three independent experiments. Figure S3. Edu pulse labelling confirms migration of irradiated S-phase cells into the G2 within 4 h post IR. Two parametric FACS histograms for (A) SY5Y-TR and (B) SY5Y-TRA cells treated with TET/NGF and measured at 0 or 4 h post IR. Figure S4. (A) EGFR phosphorylation is induced by EGF in A549 cells with wild-type EGFR, while H1975 cells bearing an activating mutation in the EGFR receptor present with EGFR auto-phosphorylation. (B, C) EGFR activation by its ligand, EGF, does not affect the G_2_-checkpoint upon irradiation. Analyses of the mitotic Index of (A) A549 and (B) H1975 after irradiation with 1 Gy and 4 Gy. Error bars show the SEM from three independent experiments. Figure S5. Western Blots corresponding to Figure 3A including molecular weight markers. Figure S6. Densitometric analyses of aggregated Western Blot data (*n* = 3) from Figure 5A. Left: SY5Y control cells (TR) or SY5Y cells with inducible expression of TrkA/NTRK1 (TRA) were used. Right: Kelly control cells (TR) or Kelly cells with inducible expression of TrkA/NTRK1 (TRA) were used. Cells were treated with TET/NGF (+) or not (−) and protein levels were determined by Western Blotting after irradiation at time points indicated. Protein expression was normalized to β-Actin. Figure S7. Impact of Loxo101 (left), ATMi (KU55933, middle) and ATRi (VE821, right) treatment on IR induced expression of pATM, pATR, pChk1 and pTrkA in SY5Y-TRA cells with (+TET/NGF) or without (-TET/NGF) activation and expression of TrkA. Notably, KU55933 and VE821 are known to inhibit downstream signaling (p-Chk1) rather than target phosphorylation. Samples were treated with inhibitors 1 h prior to IR and collected 1 h after IR at doses indicated. Figure S8. Western Blots corresponding to Figure 5 including molecular weight markers and densitometry readings. Values were either normalized to b-actin or untreated control as indicated.
