# Supplementary material for: NTRK1/TrkA Activation Overrides the G2/M-Checkpoint upon Irradiation
Source: Cancers (Basel). 2021 Nov 30;13(23):6023. doi: 10.3390/cancers13236023 (PMC8657035; doi:10.3390/cancers13236023)
Supplement: Supplementary file 1 [file cancers-13-06023-s001.zip › cancers-1455547 supplementary figure.pdf]

Supplementary Figures to : NTRK1/TrkA activation overrides the G_2/M-checkpoint upon irradiation

Christina Hassiepen, Aashish Soni, Ines Rudolf, Vivian Boron, Sebastian Oeck, George Iliakis and Alexander Schramm

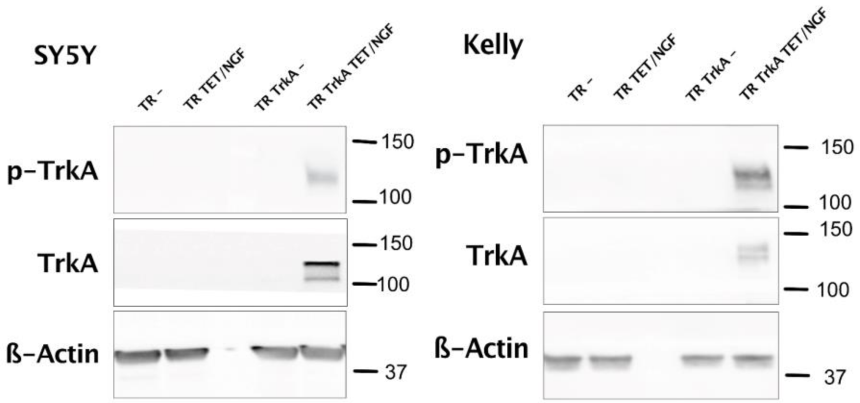

Figure S1. Western Blots corresponding to Figure 1C and 1D including molecular weight markers.

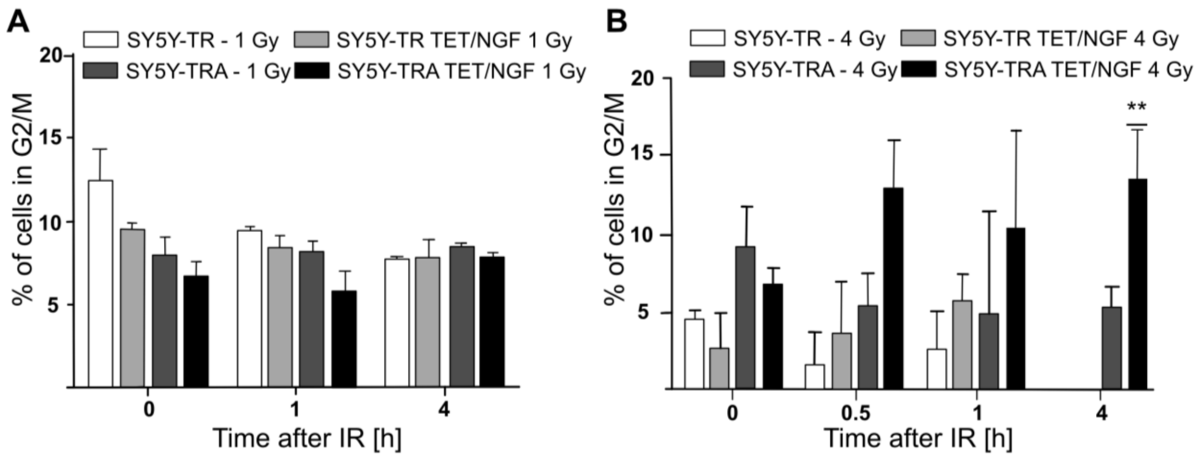

Figure S2. Activation of TrkA/NTRK1 increases the fraction of cells in G2/M phase after high-dose irradiation (4 Gy) in SY5Y-TRA. The cell cycle was analyzed by FACS and PI-staining in SY5Y cells with and without TET/NGF treatment. Cells were irradiated with [A] 1 Gy or [B] 4 Gy. Error bars show the SEM from three independent experiments.

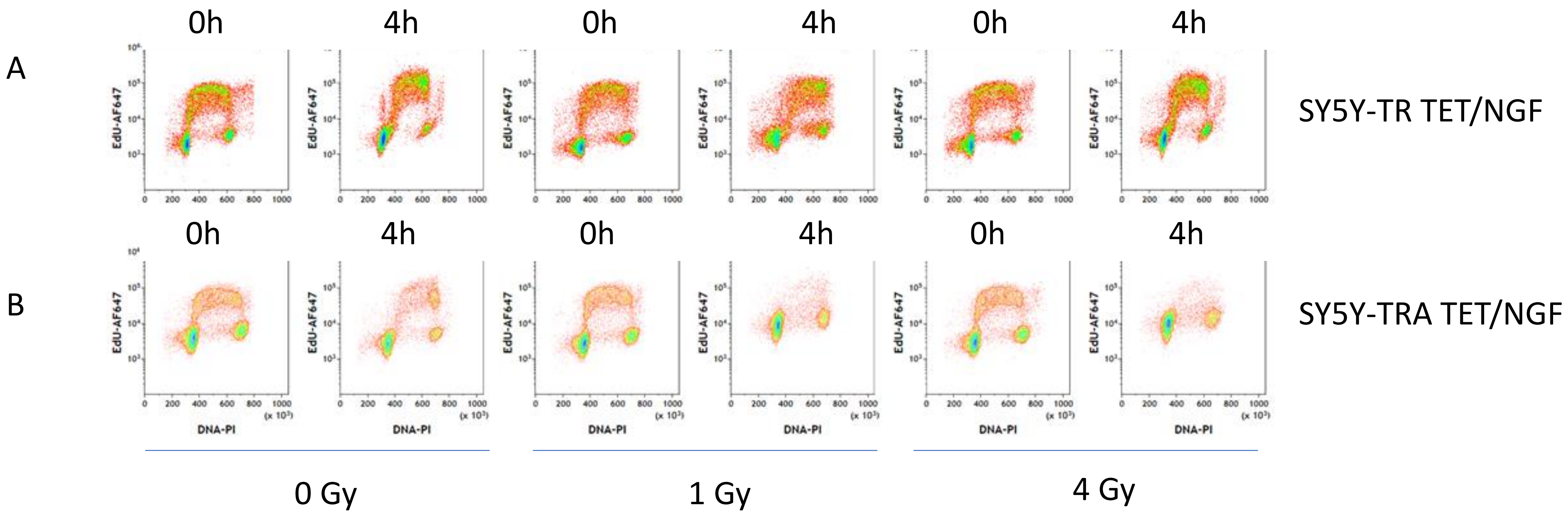

Figure S3. Edu pulse labelling confirms transition of irradiated S-phase cells into the G2 phase within 4 h post IR. Two parametric FACS histograms for (A) SY5Y-TR and (B) SY5Y-TRA cells treated with TET/NGF and measured at 0 or 4 h post IR. Cells were pulse-labeled with 10 µM of 5-Ethinyl-2´-deoxyuridine (EdU) for 15 min and then irradiated as indicated. DNA was stained using Propidium iodide (DNA-PI) plus RNAase solution for 15 min at RT.

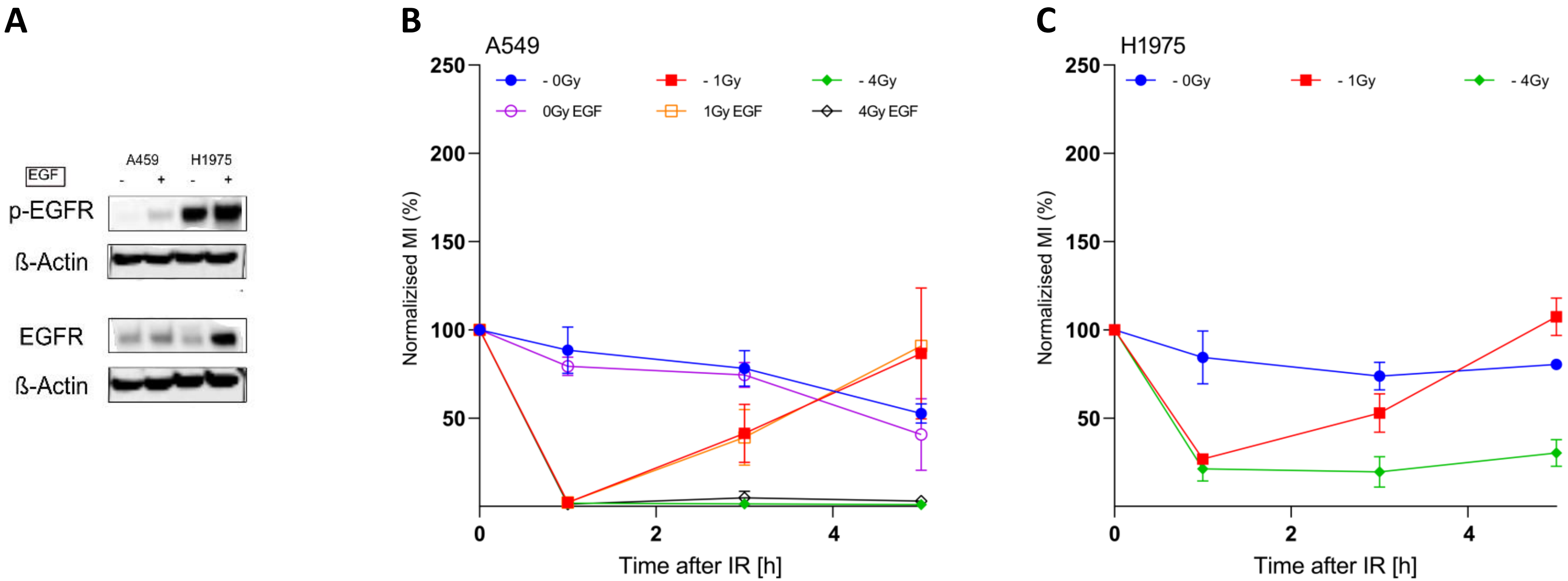

Figure S4. (A) EGFR phosphorylation is induced by EGF in A549 cells with wild-type EGFR, while H1975 cells bearing an activating mutation in the EGFR receptor present with EGFR auto-phosphorylation. (**B, C**) EGFR activation by its ligand, EGF, does not affect the G2-checkpoint upon irradiation. Analyses of the mitotic index (MI) of [A] A549 and [B] H1975 after irradiation with 1 Gy and 4 Gy. Error bars show the SEM from three independent experiments.

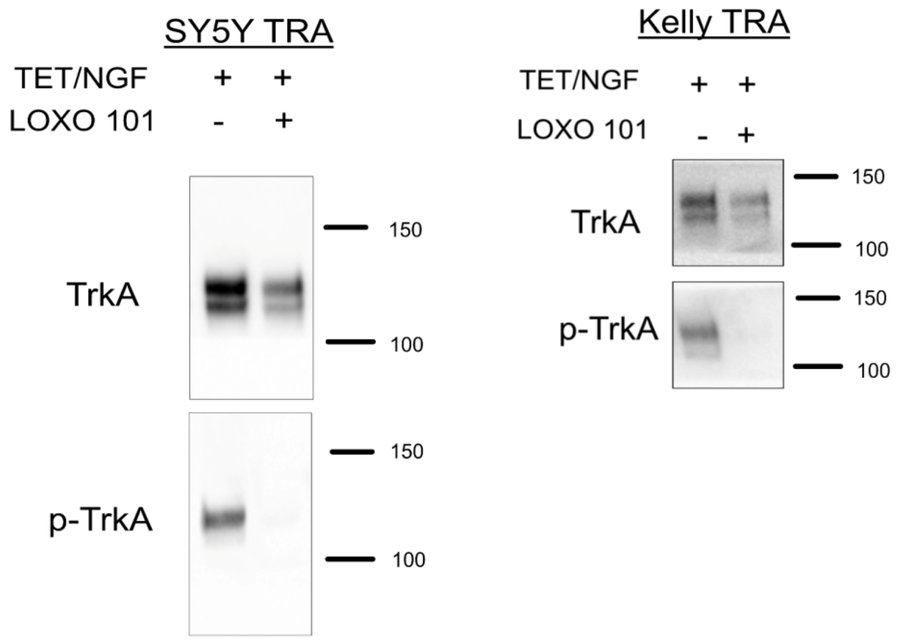

Figure S5. Western Blots corresponding to Figure 3A including molecular weight markers.

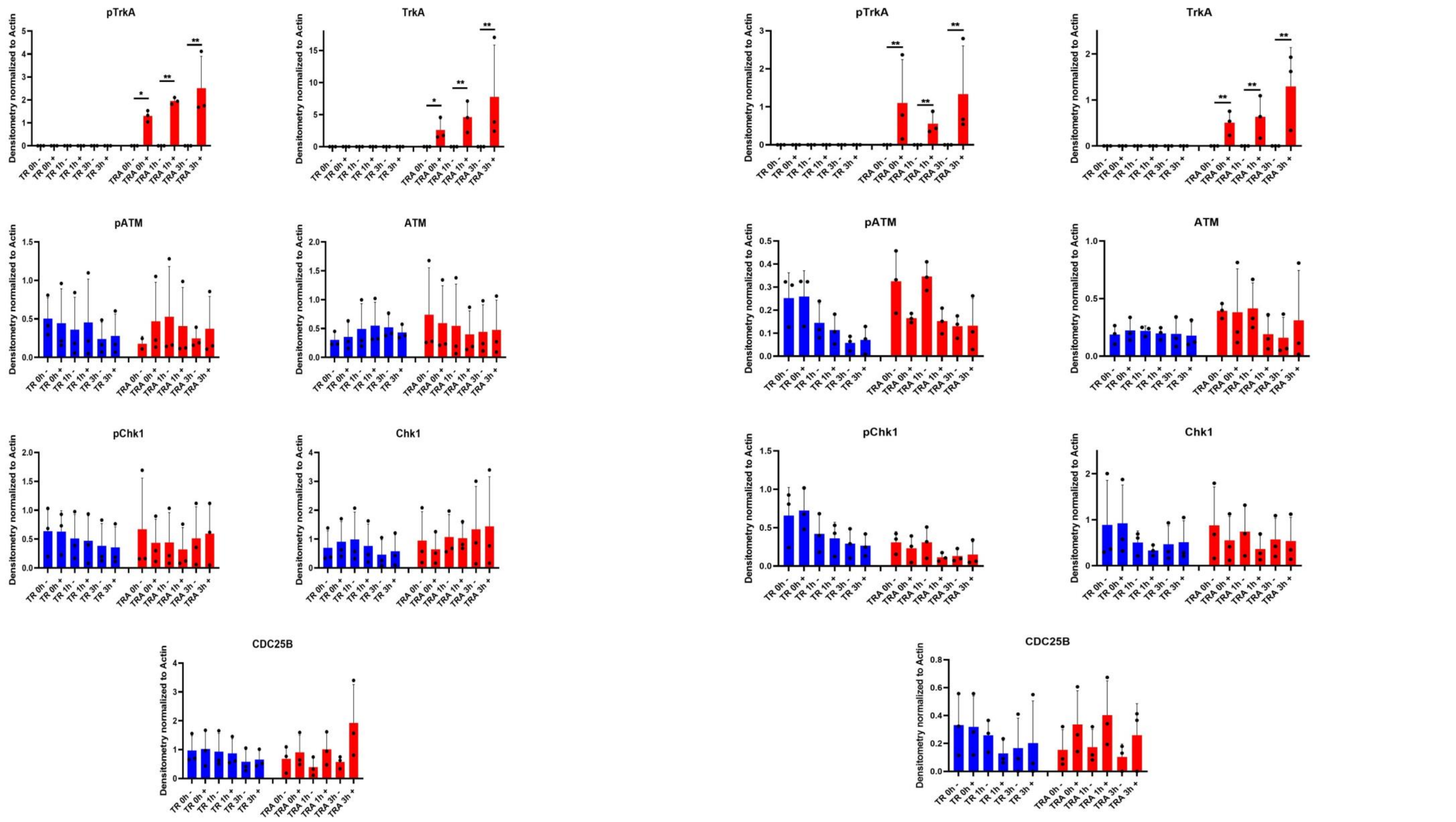

Figure S6. Densitometric analyses of aggregated Western Blot data (n=3) from Fig. 5A. Left: SY5Y control cells (TR) or SY5Y cells with inducible expression of TrkA/NTRK1 (TRA) were used. Right: Kelly control cells (TR) or Kelly cells with inducible expression of TrkA/NTRK1 (TRA) were used. Cells were treated with TET/NGF (+) or not (-) and protein levels were determined by Western Blotting after irradiation at time points indicated. Protein expression was normalized to beta-Actin.

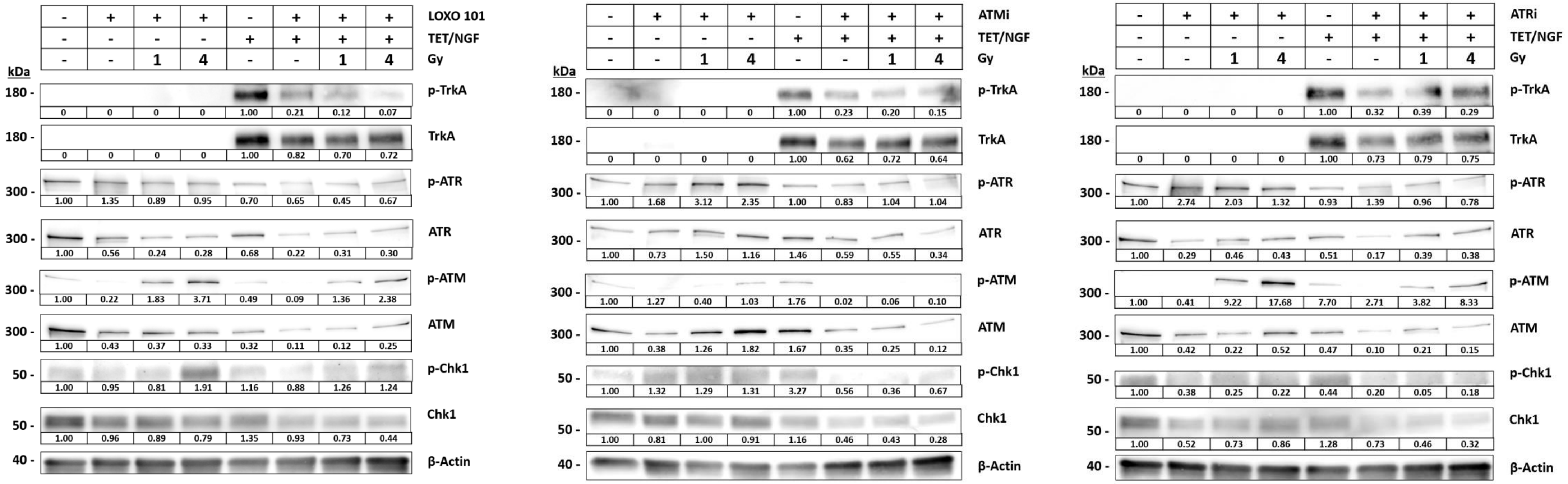

Figure S7. Impact of Loxo101 (left), ATMi (KU55933, middle) and ATRi (VE821, right) treatment on IR induced expression of pATM, pATR, pChk1 and pTrkA in SY5Y-TRA cells with (+TET/NGF) or without (-TET/NGF) activation and expression of TrkA. Notably, KU55933 and VE821 are known to inhibit downstream signalling (p-Chk1) rather than target phosphorylation. Samples were treated with inhibitors 1h prior to IR and collected 1h after IR at doses indicated.

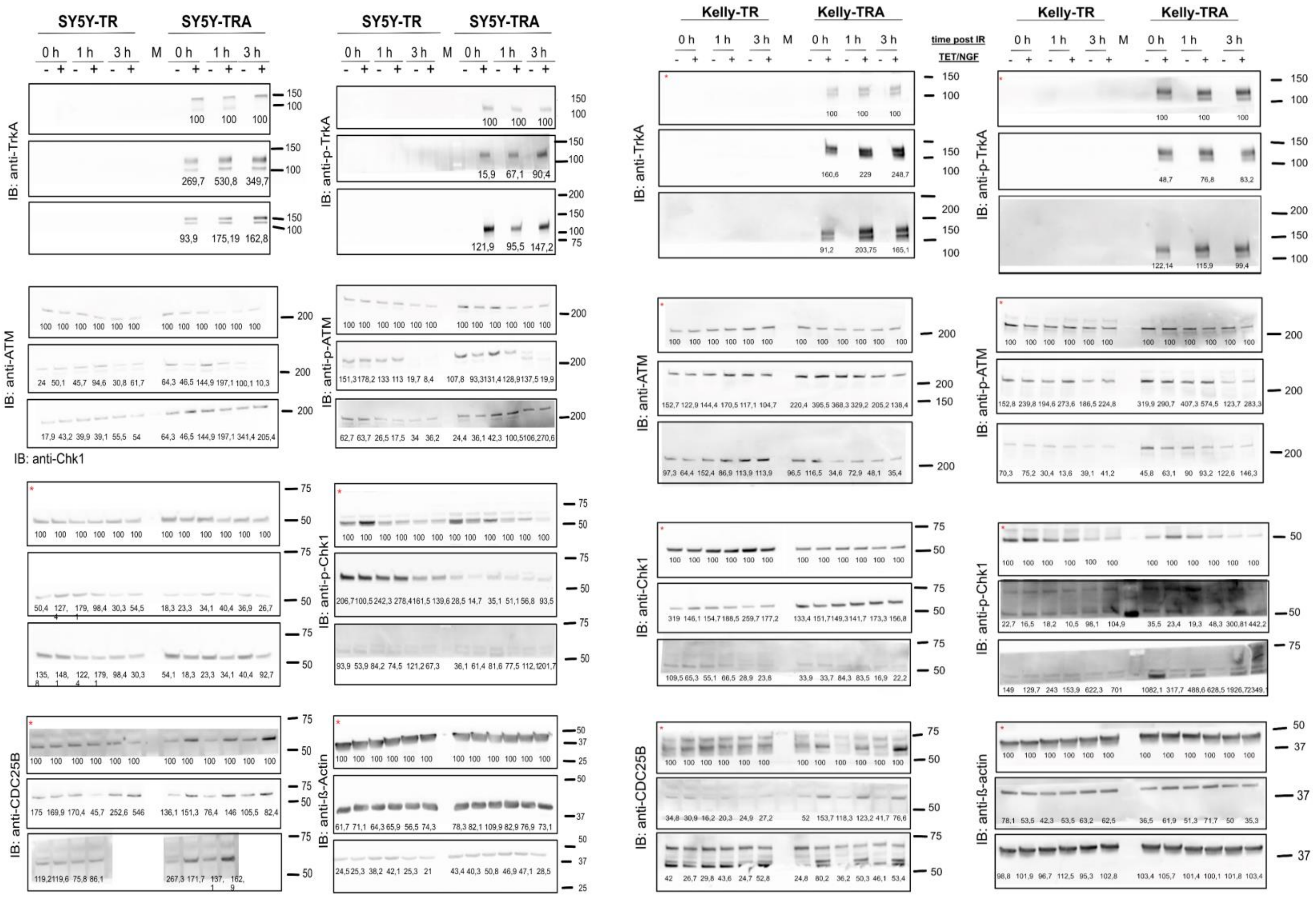

Figure S8. Western Blots corresponding to Figure 5 including molecular weight markers and densitometry readings. Values were either normalized to β-actin or untreated control as indicated.
